# LEVERAGING THE COMMUNITY AS PARTNERS TO ADVANCE CANCER RESEARCH IN SOCIALLY DIVERSE OLDER ADULT POPULATIONS

**DOI:** 10.1093/geroni/igad104.0752

**Published:** 2023-12-21

**Authors:** Shakira Grant

**Affiliations:** United States Congress, Washington, District of Columbia, United States

## Abstract

Older Black adults with cancer suffer a “double disadvantage” to their health due to the compounding effects of ageism and racism. This “double disadvantage” further fuels the disparities in cancer-related mortality observed for this population. For example, multiple myeloma (MM) is an incurable and debilitating blood cancer that predominantly affects older Black adults. Despite overall improvements in MM-related survival observed following the introduction of novel therapies, older Black persons with MM continue to die at twice the rate of their White counterparts. This survival gap is largely driven by differential healthcare access and disappears when older Black adults are given equal access to care. Using MM as a chronic disease model, we are partnering with a community advisory board to design and pilot test a community lay navigator program that seeks to address some of the upstream determinants of healthcare access that limit how older Black adults newly diagnosed with MM perceive the need for, seek, reach and engage with the healthcare system. Through our foundational qualitative work focused on older North Carolinians (patients and caregivers) affected by MM, we have identified the following multilevel (individual, interpersonal, and community level) intervention targets: trust, knowledge and health beliefs, availability and adequacy of social support, and patient empowerment. In addition, we are leveraging the resources of an established core (Rapid Case Ascertainment) housed within our Comprehensive Cancer Center and linked to the statewide N.C. Central Cancer Registry to facilitate early identification of eligible participants (within 4-8 weeks of diagnosis) and patient outreach.

